# Artificial Intelligence and Medical Humanities

**DOI:** 10.1007/s10912-020-09636-4

**Published:** 2020-07-11

**Authors:** Kirsten Ostherr

**Affiliations:** Medical Humanities Program and Department of English, Rice University, 6100 Main St., MS-30, Houston, TX 77005 USA

**Keywords:** Digital health, Natural language processing, Narrative medicine, Social determinants of health, Health technology, Big data

## Abstract

The use of artificial intelligence in healthcare has led to debates about the role of human clinicians in the increasingly technological contexts of medicine. Some researchers have argued that AI will augment the capacities of physicians and increase their availability to provide empathy and other uniquely human forms of care to their patients. The human vulnerabilities experienced in the healthcare context raise the stakes of new technologies such as AI, and the human dimensions of AI in healthcare have particular significance for research in the humanities. This article explains four key areas of concern relating to AI and the role that medical/health humanities research can play in addressing them: definition and regulation of “medical” versus “health” data and apps; social determinants of health; narrative medicine; and technological mediation of care. Issues include data privacy and trust, flawed datasets and algorithmic bias, racial discrimination, and the rhetoric of humanism and disability. Through a discussion of potential humanities contributions to these emerging intersections with AI, this article will suggest future scholarly directions for the field.

## Introduction

Until recently, the application of artificial intelligence (AI) in healthcare was a source of much speculation but little action. However, since IBM began attempting to develop healthcare applications for its “Watson” AI in 2015 (Lohr 2015; Strickland 2019), uses of AI in medicine have become tangible in a range of fields. While surveys of the industry fail to yield a single definition of AI, it is generally considered to refer to “mathematical algorithms processed by computers” that “have the ability to learn” (Zwieg, Tran and Evans 2018). Defining AI as “a set of technologies that allow machines and computers to simulate human intelligence” (Wang and Preininger 2019), clinical researchers frequently compare AI to human performance as a means of validation. Results favoring the algorithms in fields such as dermatology and radiology have provoked anxiety about job displacement in the clinical specialties that cognitive machines are expected to replace (Budd 2019). More optimistic researchers (Topol 2019; Insel 2019; Israni and Verghese 2019) have argued that AI will enhance the role of physicians, augmenting their capabilities and increasing their availability to provide empathy and other uniquely human forms of care to their patients. Regardless of their viewpoint on the desirability of AI in medicine, researchers on both sides of the debate agree that AI poses several fundamentally new challenges, due to the low degree of transparency and high degree of autonomy of “black box” AI algorithms.

Surrounding these debates are a set of practical and ethical questions about the human contexts of AI in healthcare, including issues of data privacy and security, informed consent, risk and liability, professional expertise and training, explainability of results, flawed, biased, or incomplete datasets, and unequal access to the benefits of the technology. While some of these concerns might also pertain to other domains of healthcare, this article will emphasize the challenges posed by two distinct features of AI: its voracious and indiscriminate appetite for data (both clinical and metaclinical^1^), and its purported ability to simulate human qualities. Many analyses of AI in healthcare attempt to define what is irreducibly “human” about experiences of illness and healing. The unique human vulnerabilities experienced in the healthcare context raise the stakes of new data-driven technologies such as AI, and the human dimensions of the concerns surrounding AI in healthcare have particular significance for research in medical/health humanities. This article will explain four key areas of concern relating to AI and the role that medical/health humanities research can play in addressing them: definition and regulation of “medical” versus “health” applications; social determinants of health; narrative medicine; and technological mediation of care. Through a discussion of potential humanities contributions to these emerging intersections with AI, this article will suggest some future scholarly directions for the field.

## Terminology and regulation: “medical” versus “health” data and apps

Artificial intelligence as applied across domains of human endeavor has been the subject of humanities research in disciplines including philosophy (Bostrom 2003; Gunkel 2012; Mittelstadt et al. 2016), science and technology studies (Wilson 2010; Kline 2011), and media studies (Papacharissi 2019; Guzman and Lewis 2019), and a significant body of cross-disciplinary work on algorithmic bias has appeared in the past few years (O’Neill 2016; Broussard 2018; Noble 2018; Eubanks 2018; Benjamin 2019). Looking specifically at healthcare applications, AI research has developed in computer science, medical informatics, legal studies, bioethics, and across medical specialties (Obermeyer and Emanuel 2016; Watson et al. 2019; Lin et al. 2019). For the field of medical/health humanities, AI raises a unique set of issues that highlight a central tension in the field’s evolving identity and self-definition. As Jones, Wear, and Friedman (2016) have demonstrated, since its founding in the 1970s the field of medical humanities has grown to encompass far more fields of practice and domains of human experience than the term “medical” implies. Therefore, Jones, Crawford et al. (2010), and others argue that the field should more accurately be called “health humanities” to signal an expanded view of health as influenced by more than medical interventions alone, and a recognition that health professions include more fields of expertise than those of medical doctors alone. However, when considering humanities-based research in AI, the distinctions between “health” and “medicine” pose further challenges that highlight the continued relevance of both terms for this field.

The development of AI applications for healthcare is part of a larger trend toward “digital health” practices that draw from data generated both within and beyond clinical settings. Importantly for the purposes of this article, the regulatory practices that govern the collection, storage, and analysis of data produced inside of formal clinical settings are fundamentally different from those governing data created outside of clinical settings. These differences have profound consequences for humanities research on data-driven health and medicine. Activities that take place in spaces traditionally understood as “medical,” such as hospitals and physician offices, are governed by law such as the Health Information Portability and Accountability Act of 1996 (HIPAA) and the U.S. Food and Drug Administration (FDA) premarket review process (HHS 2015; FDA 2019a). HIPAA is meant to provide safeguards for protected health information (PHI) that is personally identifiable. This law applies to “covered entities,” including doctors, hospitals, pharmacists, health insurers, and health maintenance organizations (Hoffman 2016: 73) that are traditionally involved in medical encounters. The HIPAA Security Rule has a narrow scope that excludes a large percentage of entities that handle personal health information, including websites where consumers enter health-related search terms, purchase non-prescription medications, or share personal narratives about health experiences. In sum, HIPAA covers PHI in narrowly defined “medical” contexts, and excludes broadly construed “health” contexts from regulation.

Similarly, the FDA premarket review process regulates medical devices, not health devices. Historically, the FDA’s Center for Devices and Radiological Health limited its purview to technologies used in clinical settings such as x-ray or CT scanners to ensure safe levels of radiation exposure. Recently, the agency expanded its scope through the category “Software as a Medical Device (SaMD)” to include some smartphone apps (FDA 2018b), and the agency has issued a proposed regulatory framework for AI/ML-based SaMD (FDA 2019c; 2020). In determining whether an app is subject to FDA review, the defining question is whether it claims to diagnose or treat a disease or condition. If an app makes a medical claim, such as the ability to calculate a drug dose, sense glucose, or detect arrhythmia, it will be subject to FDA regulation as a medical device (Cortez, Cohen, and Kesselheim 2014; Elenko, Speier, and Zohar 2015; FDA 2018a). Here, representation matters a great deal. The claims made in a company’s marketing materials can determine whether they are regulated or not, as exemplified by the case of the genetic testing company 23andMe’s conflict with the FDA when it advertised its product’s ability to predict and prevent disease (Seife 2013). Conversely, if an app only claims to support “health and wellness” through purportedly “low-risk” activities and information such as calorie tracking or breast-feeding tips, it will not be regulated by the FDA (FDA 2018a). Both health and medical apps may use AI/ML, but only those that make medical claims will be subject to FDA regulation. While other regulations exist that impact the uses of data and devices in digital health, the examples of HIPAA and FDA review of “Software as a Medical Device” together highlight the significance of the distinction between “health” and “medical” terminology and regulations. The existing regulatory framework leaves open a major loophole allowing technology companies such as Facebook, Google, and many others that are not “covered entities” to use websites or apps that are not regulated as SaMD, yet nonetheless capture, mine, and monetize sensitive personal health data from their users. This model relies on an outdated view of biomedical technologies as accessible only to experts operating inside of traditional medical contexts such as hospitals. Recent patient-driven movements such as Quantified Self (Neff and Nafus 2016) and #WeAreNotWaiting (Lee, Hirshfeld, and Wedding 2016) have demonstrated the potential gains to be made by redefining the sites and sources of medical expertise. Scholarship in health humanities on medical paternalism, the history of technology, and the ethics of self-experimentation can provide valuable critical frameworks for understanding these shifting power relations.

Moreover, the prevailing, narrow definition of medical devices and data is being challenged by efforts to identify “digital biomarkers” that use smartphone sensors as proxies for physiological measurements. As “consumer-generated physiological and behavioral measures collected through connected digital tools” (Wang, Azad, and Rajan 2016), digital biomarkers are the product of AI-interpreted health data from unregulated, consumer-facing sensors. Examples include the use of smartphones for continuous sound collection to enable automated cough detection, analyzed by AI/ML to detect symptoms of respiratory disease (Kvapilova et al. 2019), or the use of smartphone accelerometer or gyrometer data to detect gross motor function for AI/ML analysis of Parkinson disease severity (Zhan et al. 2018). Following the logic that, “Data becomes a digital biomarker when a relationship is drawn to a health-related outcome” (Wang, Azad, and Rajan 2016), companies are also working to use mobile engagement data such as texting and calling patterns, passively collected through a user’s smartphone, as a digital biomarker for mental health. Others aim to use step counts as a digital biomarker for medication adherence. Scholars working on the social and cultural dimensions of mental health might provide valuable context and nuance for technology companies seeking to correlate texting habits with mental health, by highlighting the ways that sociality occurs at the intersection of many forces that shape identity and self-expression, including race, gender, sexuality, age and more. Disability scholars might further contribute perspectives on the norms that are enforced by the treatment of mobility data as a biomarker.

The use of digital biomarkers to improve patient outcomes is seen by many clinicians as the ultimate goal of digital health (Coravos, Khozin, and Mandl 2019), further complicating the question of where the “medical” sphere ends and “health” begins. The existing biomedical regulatory framework underestimates the significance of metaclinical data for representing and shaping patient experiences in daily life. For humanities scholars, attention to the nuanced and shifting borderlines between “health” and “medical” devices and data through close analysis of the images, texts, and contexts that give meaning to these technologies can illuminate the contents of these two realms and the intersections of rhetorical and regulatory power therein.

## Social determinants of health

As the distinctions between “health” and “medical” data and devices suggest, the kinds of data that technology companies can mine about purchase histories, entertainment habits, social connections and geospatial mobility bear little resemblance to the kinds of data that medical doctors and researchers have traditionally used to diagnose disease. Unlike a blood glucose reading or an EKG, data from metaclinical sites like Facebook or Amazon are not sensed directly from the body, yet they do have the potential to shed light on factors that influence health (Ostherr 2018c). The term “social determinants of health (SDOH)” is defined by the World Health Organization as “the conditions in which people are born, grow, work, live, and age, and the wider set of forces and systems shaping the conditions of daily life” (World Health Organization n.d.). Researchers in medical humanities, public health, and social work (Mol 2008; Ford and Airhihenbuwa 2010; Clarke, Ghiara, and Russo 2019) have long recognized that these factors play a more significant role in individual and societal health and well-being than medical interventions or genetics alone (Mokdad et al. 2004; Graham and Bernot 2017). However, as factors that are difficult to quantify, and even harder to develop medical or pharmacological interventions to fix, SDOH have not been the focus of health technology investment until recently (Magnan 2017; Hamm 2019). Shifts in the perceived value of SDOH for healthcare present an opportunity for health humanities scholars to intervene in debates about the interpretation and significance of social and cultural context for data-driven healthcare.

Healthcare policy reform brought SDOH to the attention of the corporate healthcare sector when the Affordable Care Act (ACA) was passed in 2010, establishing “value-based care” as the new benchmark for provider payment based on quality, rather than quantity of care (Centers for Medicare and Medicaid Services 2019; Abrams et al. 2015). Under the ACA, provider reimbursement would shift toward metrics based on patient outcomes instead of the existing fee-for-service model. In the new framework, “population health” and specifically, social determinants of health, became priorities for healthcare systems with new incentives to acknowledge that critical metrics for payment, such as hospital readmission rates, were directly influenced by factors beyond the clinic walls. Therefore, providers would need to address SDOH to improve health outcomes, particularly among their most vulnerable patients. This new focus brought both preventive care and population-level concerns under the purview of medicine, yet the wide range of variables that define and shape “health” pose a significant challenge for medical doctors aiming to impact factors beyond the scope of their clinical practice (National Academies of Sciences, Engineering, and Medicine 2019).

The Health Information Technology for Economic and Clinical Health (HITECH) provisions of the American Recovery and Reinvestment Act of 2009 incentivized clinicians to participate in a broader societal shift toward digitization and “datafication” of all aspects of life, including health. Datafication has been broadly defined as “the conversion of qualitative aspects of life into quantified data” (Ruckenstein and Schüll 2017), and scholars have noted that this shift entails the commodification and monetization of health through new processes of value creation and extraction (van Dijck 2014). The transition from paper-based to electronic health record (EHR) systems created vast databases of procedure codes and billing data (Hsiao and Hing 2014). Although they were not originally designed to facilitate research or capture SDOH, these data sets are now seen as potential sources of guidance for interventions to manage patient risk, when mined by analytics programs that claim to identify signals in these noisy datasets. Yet, because EHRs do not typically capture SDOH data in sufficient detail to be useful for predictive modeling (Hatef et al. 2019), and because SDOH are often less amenable to datafication than biometric indicators, healthcare systems are beginning to utilize AI systems to fill in missing links in social determinants data.

Since 2018, AI for healthcare has been the largest market for investment among all AI business sectors (Day and Zweig 2019). Companies offering AI products for healthcare, such as Evidation, Welltok, Jvion, and Notable Health construct algorithms that use SDOH data to model patient risk profiles (Allen 2018). Many of these AI systems are trained on “lifestyle” and consumer data scraped from the web along with profiles compiled by data brokerage firms such as LexisNexis and Acxiom that include criminal records, online purchasing histories, education, accident reports, income, current and previous address, motor vehicle records, neighborhood and household characteristics, information on relatives and associates, voter registration, and hundreds of other types of data (LexisNexis 2017a; LexisNexis 2017b; Axciom 2018). Large hospital systems such as the Mayo Clinic, Intermountain Health, and the Cleveland Clinic are using these kinds of AI systems to combine data on “thousands of socioeconomic and behavioral factors” (Jvion n.d.) with the individual patient’s clinical history to guide personalized interventions and manage “risk trajectories.”

Widespread recognition of the importance of SDOH aligns with calls from humanities-trained scholars for medicine to adopt a more holistic approach to healthcare by considering the ways that social and structural inequalities impact health outcomes (Petty, Metzl, and Keeys 2017). Yet, the movement of AI into healthcare also poses troubling questions about the sources, uses, and interpretation of socioeconomic and behavioral data meant to guide the algorithms of care. Scholarship analyzing the entanglements of data and culture (Gitelman 2013) raises the fundamental question of whether datafication of social aspects of health is even possible, or desirable, particularly in light of the reductive tactics of many data mining enterprises.

While the code and data sources of AI companies are treated as proprietary trade secrets, the practices of the data brokers who supply modeling information to the industry are described in their marketing materials and provide insights into the logic governing SDOH data mining. For instance, Axciom describes how it helped a “leading health insurer” identify “specific segments of its customer base” including “prospects most likely to respond favorably” to a new wellness program to increase individual policyholder “engagement and loyalty” (2019). In light of the emphasis on “return on investment” in promotional materials for these products, this description seems to imply that the insurer did not use the SDOH data mining tool to provide more wellness benefits to the neediest patients, but instead, to attract the lowest risk, highest profit customer segment. Similarly, LexisNexis notes in its SDOH product advertising, “Liens, evictions and felonies indicate that individual health may not be a priority” (2018). A critical race theory approach to public health (Ford and Airhihenbuwa 2010) would view this statement as indicating the need for additional resources to engage and support the affected community. Instead, the implication here seems to be that LexisNexis data mining tools can guide health industry clients to exclude prospective patients with undesirable histories. When the company further points out that, “Poor members of racial and ethnic minorities are more likely to live in neighborhoods with concentrated poverty” (LexisNexis 2017c), they could be highlighting the role of racial discrimination as a factor that demonstrably shapes health outcomes through structural and individual harms (Abramson, Hashemi, and Sánchez-Jankowski 2015; Luo et al. 2012; Pascoe and Richman 2009). Instead, LexisNexis seems to urge customers to utilize race as a medical risk classification, a practice that has been thoroughly critiqued by ethicists, historians, critical race and legal theorists, geneticists, and biologists (Yudell et al. 2016). Scholars working in and across these fields are well-positioned to identify and critique spurious efforts to use SDOH data in this way.

As these examples attest, the use of SDOH data acquired from sources other than the patients themselves poses the risk of reproducing the same human and structural biases that produced protected identity categories in the first place. Indeed, Obermeyer et al. (2019) recently demonstrated this problem by showing how an AI system widely used in hospitals in the United States produced racial bias when decisions were modeled on insurance claims data, while the disparities were almost entirely eradicated when the algorithm trained instead on biological data from patients. Murray and colleagues have further shown how built-in AI tools for EHRs may propagate health inequities by classifying personal characteristics such as ethnicity or religion as risk factors (2020). Humanities scholarship on ethnicity and health, or religion and health, could contribute valuable reframing of this approach to data analysis by working directly with software developers.

Taking a somewhat different approach, researchers at Facebook have emphasized the value of user-generated data by arguing that it has higher fidelity to its original source (i.e. the patient) than many traditional sources of healthcare data, because social media postings provide a direct window into a user’s social life. In an article titled, “Social Determinants of Health in the Digital Age” (Abnousi, Rumsfeld, and Krumholz 2019), Facebook’s Head of Healthcare Research and his co-authors argued that SDOH data from social networks should be combined with data from health records to improve patient outcomes. The article proposes a “granular tech-influenced definition” of SDOH that includes “numbers of online friends” as well as “complex social biomarkers, such as timing, frequency, content, and patterns of posts and degree of integration with online communities” drawn from “millions of users” (247). The authors urge readers to imagine the “richness of possible connections that can be explored with machine learning and other evolving ‘big data’ methodologies,” including topics that Facebook has already begun to explore: suicide prevention (Thielking 2019), opioid addition (Facher 2018), and cardiovascular health (Farr 2018). In light of the well-documented privacy violations committed by Facebook in the Cambridge Analytica scandal (Rosenberg and Frenkel 2018) and in relation to patient groups (Ostherr 2018a; Ostherr and Trotter 2019; Downing 2019), the company’s efforts to merge SDOH data with EHR data raise significant privacy concerns while also highlighting the need for new critical and policy frameworks that prioritize patient perspectives and health equity, rather than financial perspectives, on the value of SDOH data.

The erosion of boundaries between SDOH data from our activities on Facebook (as well as Google, Amazon, and other sites) and clinical care environments may have serious implications for patients in the future. SDOH data is inherently problematic when it comes to patient privacy, because the value of the data is dependent on its specificity – two patients with similar age, weight, race, and diagnosis but different zip codes or education levels could have very different risk profiles. Therefore, for SDOH data to be valuable, it cannot be treated in aggregate. Yet, the demonstrated ease with which purportedly anonymized health data can be reidentified (Sweeney et al. 2017; Yoo et al. 2018), shows that it is virtually impossible to protect patient privacy when mining SDOH data. As growing numbers of public-private initiatives - such as the “All of Us” research program at the National Institutes of Health (NIH 2019) - merge health records and social media data in the effort to assess SDOH, the need for multidisciplinary research that brings critical perspectives and interpretations from the humanities to data privacy and social dimensions of health will only grow.

## Narrative medicine and natural language processing

A major reason that social determinants of health data hold so much appeal for researchers is that they provide the fine-grained, nuanced descriptors that give meaning and context to individual lives. Since at least the 1970s, scholars in medical and health humanities have recognized the value of personal narratives as sources of perspective on patients’ lives, publishing accounts of the value of listening to, reading, and writing illness narratives (Ceccio 1978; Moore 1978; Peschel, 1980; Trautmann and Pollard 1982). Practitioners of “narrative medicine” have argued that patient stories are a vital component of the medical record (Charon 2006; Charon et al. 2016), and efforts should be made to include them, rather than allowing them to be replaced by generic, drop-down menu selections. On a broader scale, major health organizations around the world have begun to emphasize the need to incorporate patients’ perspectives in healthcare and research (Snyder at al. 2013). However, since the HITECH Act of 2009, the increased use of EHRs favoring quantitative data and drop-down menus over narrative text has posed a significant challenge to advocates of narrative medicine who see the patient story as central to the practices of diagnosis and healing (Patel, Arocha, and Kushniruk 2002; Varpio et al. 2015). Natural language processing (NLP), a subfield of AI, is poised to transform debates about the status of the patient narrative, both within clinical EHRs and in metaclinical ecosystems. In simple terms, NLP is a “range of computational techniques for the automatic analysis and representation of human language” (Young et al. 2018). In practice, NLP is at work anytime a Google search entry is auto-completed, Siri converts spoken words into text, or a chat-bot interprets a human user’s needs and helps them to complete a transaction. The use of NLP for healthcare is promoted in part as a method for addressing the need to represent patient perspectives in medicine by utilizing computational text mining to better integrate qualitative and quantitative data in patient records (Denecke et al. 2019). Given the subtleties entailed in representing patient narratives through diverse forms of mediation, these emerging data science methods would benefit from the insights of health humanities scholars with expertise in the interpretation of narratives in complex intersubjective, social, and cultural contexts.

In the highly structured data fields of EHRs, one particularly important section – the doctor’s note, where the closest approximation of the patient’s story resides – remains unstructured. While the non-standardized format of narrative prose makes it challenging for traditional data analytics programs to interpret and codify (Bresnick 2017), humanities scholars argue that it is precisely the nuanced, context-dependent style of the doctor’s note that can make it a valuable source of information about the patient’s perspective, lifestyle, preferences, and illness experience (Charon et al. 2016). To fulfill this function, however, at least three aspects of the EHR must change: space must be preserved or increased for open-ended text; the note must actually represent the patient’s version of the story, not only the doctor’s version of that story; and techniques (such as NLP) must be developed for interpreting potentially vast quantities of narrative in relation to equally vast quantities of numerical data. Some efforts toward this goal have taken place under the rubric of the Open Notes movement (Bell, Delbanco, and Walker 2017; Fossa, Bell, and DesRoches 2018), demonstrating how clinical free-text notes, when written with patient participation in mind, have fostered improved engagement, shared decision making, and patient-centered care.

An alternative to the EHR-centric approach proposes instead to use NLP on metaclinical data that might be interpreted and integrated into the patient’s record as a supplemental source of narrative data. NLP is seen by some clinical researchers as capable of providing access to patient perspectives by integrating analysis of their clinical records (including unstructured free text), with user-generated content from social media (such as Twitter, Facebook, Reddit, and Instagram) and online health communities (Gonzalez-Hernandez et al. 2017). When coupled with the healthcare industry’s newfound interest in social determinants of health, NLP can be seen as an essential tool for extracting data from metaclinical sources and meshing it with clinical data to produce AI-driven patient risk modeling and decision support (Denecke et al. 2019). While the privacy and health equity issues associated with social data scraping factor heavily in discussion of the ethics of accessing and utilizing these sources of data (Hargittai and Sandvig 2015), for the purposes of this section, the key question is what role might NLP (and its variants) play in shaping the future of patient participation and narrative medicine? Put differently, could NLP be marshalled by health humanists as a mechanism for restoring the patient’s voice to the center of the healthcare experience, or is it a step too far toward automation of human narratives of illness and caring?

While the value of NLP for meaningful access to patient perspectives may at first seem doubtful, it is worth considering some test cases. In a recent study, Ranard and colleagues (2016) considered whether unstructured patient reviews of hospitals on the social media platform Yelp might enhance the understanding of patient perspectives. The authors compared insights derived from Yelp with those offered by the Hospital Consumer Assessment of Healthcare Providers and Systems (HCAHPS) survey, the current standard for capturing patient feedback about U.S. hospitals. Using NLP to mine text and conduct sentiment analysis on a Yelp dataset of over 16,000 reviews of U.S. hospitals, the researchers found that the majority of reviews expressed patient and caregiver experiences that were not identified by HCAHPS domains, suggesting that Yelp provided a window into what mattered most to those reviewers, in their own words. The authors concluded, “Online platforms are democratizing in ways that answering preassigned questions can never be—because giving voice to patients also means giving them the opportunity to select the topics” (Merchant, Volpp, and Asch 2016, 2484). The scale of the Yelp dataset, combined with the personal, often emotional details included in the posts may present more credible patient perspectives than the generic HCAHPS survey ever could, even when those perspectives are unearthed through computational rather than human interpreters.

At the level of individual patient care, clinicians have also sought to mine large datasets of patient perspectives drawn from personal narratives. For example, in a study of the role of narrative in shaping patient decision-making, Dohan et al. (2016) collected stories from a hundred patients with advanced cancer. Seeking to uncover patterns that might serve as an evidence base for future patients, the researchers proposed the concept of a large-scale “ethnoarray,” similar to a genetic array, that would include patients’ demographic characteristics, clinical conditions, decisions, and outcomes. By mining large narrative datasets and representing the results in a quantitative format – a “narrative heat map” – that displayed many patient experiences in aggregate, “researchers, clinicians, patients, and caregivers [could] more easily understand how their own experience compares to the cancer journeys of others” (723). Seeing patients’ stories as valuable guidance for other patients, the researchers observed that in one case study, access to a cancer survivor’s narrative enabled a patient to reframe her approach to treatment so that it would better align with her own values and preferences. The authors explained, “From the perspective of clinical evidence, mastectomy was unnecessarily aggressive, but from a personal perspective, the procedure aligned with [the patient’s] feelings about her identity, sexuality, and sense of empowerment” (721). Echoing the findings of Ranard et al. (2016), this study concluded that narratives describing illness trajectories provided perspectives that were otherwise unavailable. The prospect of opening up access to thousands of patient stories to provide supportive evidence for a diverse range of pathways suggests the potential for NLP/AI-driven narrative medicine to humanize the practice of care.

Yet, as with SDOH data, mining patient stories with NLP – and other emerging techniques such as deep learning and neural networks – raises concerns not only about erroneous, decontextualized, and biased results, but also about trust, privacy, and security. A recent law suit filed against Google and the University of Chicago Medical Center (Schencker 2019), *Dinerstein v Google*, illustrates the privacy concern that arises when technology companies seek out clinical partners to gain access to health data for the purpose of training their AI systems (Cohen and Mello 2019). The complaint alleges that the medical center shared identifiable data from the electronic health records (EHRs) of thousands of patients who were treated at the hospital between 2009-2016. Despite being ostensibly “de-identified,” the complaint claims that those records contained time-stamps that, when combined with Google’s access to geolocation and other types of data, could easily reidentify a patient (Wakabayashi 2019). Google researchers had already publicized this work in the journal *Nature* (Rajkomar, Oren, et al. 2018), describing their methods for training their deep learning system on “the entire EHR, including free-text notes,” providing further support for the plaintiff’s complaint that privacy rules were violated when Google obtained these records. Moreover, Google had filed a provisional patent in 2017 (Mossin et al. 2017) for a proprietary EHR system that would build on the company’s mining of patient records from the hospitals in Chicago to develop and sell AI-driven predictive EHRs for commercial gain. Recent reporting on another Google AI/EHR endeavor with Ascension health system (Copeland 2019) confirms that such “breaches” are in fact part of a systematic effort to develop comprehensive digital health profiles of Google’s enormous user base.

Moreover, these patient privacy violations mirror those committed by Google’s DeepMind in 2017, when the company used patient records from the UK’s National Health Service to build risk analytics tools without patient consent (Lomas 2019). In response to a review by the U.K. Information Commissioner’s Office which found that the deal between the NHS and DeepMind broke data protection law, Google’s DeepMind team acknowledged, “There is no doubt that mistakes were made, and lessons must be learned” (Stokel-Walker 2018). Yet, the *Dinerstein v Google* lawsuit suggests that those lessons have not been learned, as Google continues to capitalize on its ability to combine non-health-related consumer data with highly sensitive medical data, without consumers’ awareness or ability to opt out. Google’s plans to develop commercial EHR software, along with its acquisition of Fitbit (Robbins and Herper 2019), raise additional concerns that patient experiences in future healthcare encounters will be shaped by AI interpretation of their digital footprints without patients’ awareness, consent, or ability to challenge those results.

Further complicating the role of NLP mining of clinical and metaclinical patient data, the work of Sweeney and colleagues (Malin and Sweeney 2004; Sweeney 2015) has shown that true de-identification of patient records is currently impossible. Yet, researchers seeking to gain clinical insights from patient narratives have noted that when a patient record is stripped of all identifying data – all PHI – that record is also stripped of all narratively meaningful information (Dohan et al. 2016; Yoo et al. 2018). Therefore, the potential gains of increased attention to patient stories through large scale text-mining methods must be understood in the context of privacy compromises that presently appear insurmountable. Yet, if the core objective of narrative medicine is for patient experiences to be better understood and incorporated into practices of care, might humanities scholars help respond to this need through alternative approaches to self-narration? If narrative understanding provides a foundation for trust, is it possible to imagine a computational approach to trust that is grounded in personal, rather than transactional, exchanges? Collaborative computer science and health humanities efforts to identify viable alternatives would open up important new avenues for research on patient narratives in healthcare. Digital humanities approaches to narrative text mining may also offer valuable methods that could be adapted for the unique contexts of medical records (Arnold and Tilton 2015).

## Technological mediation of care

For proponents of narrative medicine, patient stories promise to balance the quantitative imperative with the human contexts of illness and healing, and the tension between data and stories is central to debates about AI. Much of the publicity surrounding the adoption of artificial intelligence in healthcare has focused on its potential impact on the “humanity” of both doctors and patients, but concern over how technology mediates human interaction in medicine has a longer history. In *Medicine and the Reign of Technology* (1978), Stanley Reiser recounted how Laennec’s invention of the stethoscope in 1816 seemed to produce a technological barrier between doctors and patients that would eventually lead medicine toward quantitative epistemologies and undermine trust in the patient’s narrative as a source of clinically relevant information (Reiser 1978; 2009). Since the discovery of x-rays in 1895, the practice of medicine was further mediated by interpretation of visualizations (Lerner 1992), and subsequent generations of imaging devices have provoked debate about how health and disease are seen and understood through processes of visual mediation (Dumit 2004; Joyce 2008; Ostherr 2013). The introduction of electronic health record systems in the 1960s altered the spatial and temporal dimensions of healthcare information and communication exchange (Weed 1968), fragmenting the patient record into decontextualized data points and further mediating the doctor-patient encounter through the abstractions of procedural coding. By considering these technologies as media, that is, as interfaces through which information is coded and transformed into meaning, researchers can identify the values that are embedded in and activated by the seemingly neutral devices that populate the healthcare ecosystem. Moreover, by emphasizing the situated, sociotechnical aspects of mediation (Wajcman 2010), researchers can illuminate how the idea of machine objectivity (Daston and Galison 1992) obscures the uneven distribution of the effects of technologies such as AI across diverse populations. As AI becomes enmeshed with the internet of health and medical things, it holds the potential to mediate and thereby interpret the very definition of health and disease. For this reason, there is a need for research on the ways that AI and other cognitive technologies mediate meaning in healthcare contexts.

Depictions of the future of AI in medicine have existed far longer than any actual AI programs in medicine. Yet, the sometimes fictional nature of AI representations in no way undermines their ability to shape ideas about and experiments to develop new technologies (Kirby 2011). Popular depictions in films such as *Prometheus* (Scott 2012) and the television series *Humans* (Vincent and Brackley 2015-2018) and *Black Mirror* (Brooker 2011-present) present AI as a dehumanizing threat to medical doctors and patients alike, and Longoni, Bonezzi, and Morewedge (2019) have shown that most patients do not trust the idea of medical AI, even when actual AI systems are shown to outperform human doctors. Acknowledging this image problem, Jordan and colleagues (2018) describe the practice of technology companies hiring science fiction futurists to help imagine new directions for research and development as a form of “science fiction prototyping.” Recognizing the power of these representations to shape attitudes and investments in AI for healthcare, technology companies have developed strategic campaigns to represent a more favorable vision of AI. One strand of this marketing emphasizes augmentation of physicians through the humanistic and empathic effects of AI in medical settings, and another characterizes AI for patient-consumers in health contexts outside of medicine, where a recurring focus on disability prevails. Through these discursive framings, AI proponents engage topics that medical/health humanities researchers are well positioned to engage.

Garland Thomson (1997), Garden (2010), Banner (2017) and others have critiqued how representations of disability in popular media shape broad cultural narratives that define disability as an individualized “tragedy” to be overcome. Exemplifying this phenomenon, technology companies such as Apple, Microsoft, and Google promote AI projects to help people with disabilities “overcome” their purported limitations. The companies describe their AI as “humanistic” or “human-centered” (Menabney 2017; Ostherr 2018b), but the “human” in that framework is narrowly defined through able-bodied norms. For instance, in his TED talk on AI (2017), Apple product designer and Siri co-creator Tom Gruber celebrated how his friend Daniel, who is blind and quadriplegic, used Siri to meet women online and manage his social life through email, text, and phone, “without depending on his caregivers.” Gruber observed, “The irony here is great. Here’s the man whose relationship with AI helps him have relationships with genuine human beings. This is humanistic AI.” Crip theorists critique such individualistic framing of disability, arguing that the focus should shift from developing technologies to aide people with disabilities to reimagining the social spaces and policies that enforce norms about ability and perpetuate exclusion in everyday life (Bennett and Rosner 2019; Williams and Gilbert 2019).

Following the Siri developer’s logic, both Microsoft (Wiggers 2019) and Google (Vincent 2019) used their 2019 developer conferences to highlight their work on AI applications for accessibility. Microsoft’s “AI for Good” program aims to create “human-centered AI” through a smartphone app called “Seeing AI.” The mobile app is meant to help visually impaired people “engage more fully in professional and social contexts” through features such as “friend recognition, describing people and their emotions, using store barcodes to identify products, and reading restaurant menus out loud” (Heiner and Nguyen 2018). While these apps may bring some practical benefit to their users, they also mediate the world through filters that reflect and perpetuate normative worldviews. Moreover, these apps provide cover for the companies who create and market them as technological manifestations of the human-centered principles that they claim will govern their overall AI development strategy. The contrast is particularly evident at Google, whose “Project Euphonia” – part of their “AI for Social Good” program – aims to expand the use of voice interfaces to people with speech impairments. To do so, the training data for speech software like Google Assistant must expand the scale, range, and diversity of its samples, and Google utilized their 2019 I/O developer conference to solicit data donations from the thousands of programmers in attendance (Vincent 2019). As with many practices of contemporary technology companies, the seemingly benevolent objective of assisting people with disabilities also serves the company’s primary aim of population-scale data extraction and mining. Goggin and Newell (2005) have critiqued this type of subterfuge, arguing, “Disability is customarily invoked as a warrant for development of new technologies, from biotechnology to information and communication technologies, and ‘smart homes.’ Yet the rhetoric of such claims, their purposes, truths, and styles, are rarely analyzed and interrogated.” The researchers call for work that “examines the technology of disability in terms of its cultural and social context, constitution, and relations.” The rhetoric of “humanistic AI” affords numerous opportunities for such critical engagement, in relation to both disability and its companion, augmentation.

Former chief scientist for AI at Google’s cloud division and current Director of the Institute for Human-Centered Artificial Intelligence (HAI) at Stanford University, computer scientist Fei-Fei Li is an influential proponent of human-centered AI (2018). In her speech launching the HAI (2019), Li used healthcare as an example of the ways that AI can “augment its human counterpart.” Li depicts a hypothetical hospital emergency room scenario where an AI computer vision algorithm constantly scans a crowded waiting room to assist overburdened healthcare providers, interpreting the facial expressions and idiosyncratic communication styles of the assembled patients and their companions. As Li (2019) describes the scene, the AI-powered triage system

can speed up preliminary diagnostics by understanding the context of limp or slurred speech, cross-referencing its observations with the patient’s medical records. Imagine that it can make educated guesses about the patient’s emotional state based on their face and posture. And imagine, it can keep an artificial eye and ear on every patient while they wait, watching for changes of their medical and emotional state, and keeping the clinician up-to-date. And imagine, it all works in real time for everyone in the ER, the effect would be transformative. Clinicians would remain face-to-face with their patients but with less stress and greater focus. Each interaction would begin with the insightful head start. And in the ER, saving time is often saving lives.

This vision of AI extends the framework of technological mediation in healthcare to perpetual and pervasive surveillance, cross-referencing patient observation not only with their EHR but also with their entire Google data profile. Considered in light of the *Dinerstein v Google* lawsuit discussed above (Schencker 2019), this vision of AI-driven healthcare raises serious concerns about privacy, bias, and misinterpretation of SDOH contextual cues. In addition, this description exemplifies the concept of AI as technological mediation: every expression, behavior, and movement of the patient is sensed and interpreted through algorithms fed by sources from within and beyond the medical setting. The clinician sees the patient mediated through this matrix of data, with all of the attendant encodings of that patient’s digital self. Proponents argue that this type of machine vision will transcend the biases of human clinicians (Rajkomar, Dean, and Kohane 2019; Rajkomar, Hardt, et al. 2018), but the risk of submerging discriminatory interpretations under layers of code in this vision of medical augmentation through AI instead poses the greater threat of compounding harms by making them “invisible.”

For example, when Google Photo’s image-labeling algorithm classified black people in photographs as “gorillas” (Barr 2015), the company apologized, pointed to the limitations of machine learning and, in a misguided approach to addressing the problem, removed the label of “gorilla” from the system, thereby rendering its racism invisible. Several years after the incident, the image search algorithm still excluded the search term “gorilla,” along with “chimp,” “chimpanzee,” and “monkey” (Simonite 2018). Google also excluded the categories “African American,” “black man,” and “black woman” from their Photo labelling categories. Research by Klare et al. (2012), Buolamwini and Gebru (2018), and Snow (2018) has further shown how computer vision (a form of AI) can lead to biased results such as misclassifying the gender of darker-skinned people in automated facial analysis algorithms and datasets. Building on studies that show how racial bias is embedded in natural language processing programs (Bolukbasi et al. 2016; Caliskan, Bryson, and Narayanan 2017), this body of research demonstrates the disastrous consequences that can arise from the use of algorithms to mediate decision-making in healthcare (Benjamin 2019). The known problems with bias in computer vision and NLP raise serious concerns about racial, gender, class, and other forms of discrimination in the hypothetical AI-augmented emergency room of the future.

Yet, the lure of humanistic AI rhetoric is powerful. Clinician researchers frequently cite the ability of AI systems to outperform human doctors (Titano et al. 2018), thereby improving patient care. Medical doctor and Google-trained AI researcher Eric Oermann insists that, “bringing more machines into medicine […] will let physicians focus more on patients” (Miller 2019). Israni and Verghese (2019) elaborate on this perspective, asking,

Could AI help clinicians deliver better and more humanistic care? Beyond easing the cognitive load and, at times, the drudgery of a busy practice, can AI help clinicians become better at being human? The desirable attributes of humans who choose the path of caring for others include, in addition to scientific knowledge, the capacity to love, to have empathy, to care and express caring, to be generous, to be brave in advocating for others, to do no harm, and to work for the greater good and advocate for justice. How might AI help clinicians nurture and protect these qualities? (29)

Through this rhetoric of humanism, warnings that technology can lead to a dehumanizing loss of empathy are transformed into promises of personalized medicine mediated by technology (Darcy, Louie, and Roberts 2016). While patients and doctors alike might agree that medicine would benefit from more personal and empathic care, the idea that AI would allow doctors more time with their patients instead of filling their time with more tasks seems doubtful. Yet, the exact role that AI will play in mediating doctor-patient relationships remains to be determined, and therefore, critical analysis of AI as a technological mediation of care could influence future developments in the field. At least three levels of mediation should be considered. First, researchers should explore how representations of AI in healthcare shape ideas about disability, accessibility, augmentation and empathy. Second, they should identify how definitions of health, illness, intervention and care are filtered through the lens of AI. And third, they should expose how AI mediates and renders invisible discriminatory interpretations of identity as it constructs and analyzes user profiles. Humanities methods for interpreting and explaining how AI intervenes in medical ways of seeing and knowing will be vital for tracking the transformations that are likely to occur as this new technology becomes fully integrated into clinical ecosystems.

## Conclusion

AI is an evolving technology with many entanglements that offer productive sites for health humanities research. Healthcare systems, big technology companies, pharmaceutical firms, insurance payors, electronic health record vendors, patient networks, regulatory agencies, governments, scholars, critics, and AI developers themselves are in the process of determining how these cognitive systems will change how we live and die. The potential benefits of AI for diagnosis, treatment, and drug discovery generate optimism and hope for new knowledge and better patient outcomes. The potential harms of algorithmic bias and further dehumanization of healthcare generate calls for transparency and accountability in how these systems are deployed. Amidst these debates, humanists can contribute expertise in the language and contexts of “health” and “medicine,” social determinants of health, narrative medicine, and technological mediation. In addition, further scholarship on AI and disability, personal genome sequencing and enhancement, intersections of race, gender, and sexuality in technology development, and indigenous and other forms of medical epistemologies would be valuable contributions to the field.

While AI is already being utilized around the world (Feldstein 2019), the contexts for AI in healthcare must be seen through geographically specific frameworks, as the regulatory and cultural factors shaping their use vary widely by national and regional specificity. In the European Union, for example, the General Data Protection Regulation (GDPR) implemented a privacy policy in 2018 that granted rights to citizens and established rules for businesses, limiting their data-tracking scope (European Commission 2018). In the United States, the rights of the individual are enshrined in HIPAA and the Common Rule, but are poorly enforced (Tanner 2017) and do not apply to metaclinical settings. While GDPR is influencing privacy policies among many global companies, these protections are not evenly distributed around the world, as demonstrated by new efforts to bring unregulated data mining and AI to global health. One such effort is the “Precision Public Health Initiative” recently launched by the Rockefeller Foundation (2019), which aims to use artificial intelligence on diverse data from sources including social media to prevent premature deaths in India, Uganda, and eight other countries. Beyond policy differences, the global dimensions of narrative medicine vary across cultural contexts, both in the role of patient stories within healthcare practice, and in the distinct forms of knowledge that emerge from diverse medical traditions (Muneeb et al. 2017; Huang et al. 2017; Fioretti et al. 2016). Comparative studies of AI in global contexts are needed to fill this critical research gap.

In the United States, clinical spaces are filled with screens and networked computers, but consideration of how these technologies might impact the experiences of human beings in the healthcare ecosystem often occurs only after they have been fully deployed. As AI systems become further entangled with health and illness in clinical and consumer-oriented spaces of care, they extend the technological mediation of medicine while claiming to restore its humanity. However, unlike many older medical technologies such as stethoscopes or x-rays, AI is unevenly distributed across healthcare settings, and its fate in the clinical armamentarium is yet undecided. Medical and health humanities scholars must play a role in shaping the future of AI in healthcare.

## Endnotes

^1^Elsewhere I have defined “metaclinical” spaces as “those sites constituting the vast ecosystem outside of traditional clinical settings where consumer-patients engage in behaviors that may be directly or indirectly related to self-management of health and disease, whose digital traces can be captured and incorporated into data-driven frameworks for health surveillance and intervention” (Ostherr 2019).
